# Comparison of Opioid Consumption During Paediatric Anaesthesia with and Without a Mandatory Protocol: A Retrospective Cohort Study

**DOI:** 10.3390/jcm14217481

**Published:** 2025-10-22

**Authors:** Maciej Kaszyński, Barbara Stankiewicz, Aleksandra Kalicka, Karolina Mikołap, Monika Olszanecka, Zuzanna Rybka, Paweł Witt, Marek Darowski, Izabela Pągowska-Klimek

**Affiliations:** 1 Department of Paediatric Anaesthesiology and Intensive Care, Medical University of Warsaw University Clinical Centre, 02-091 Warsaw, Poland; 2 Department of Modeling and Supporting of Internal Organs Functions, Nalecz Institute of Biocybernetics and Biomedical Engineering, Polish Academy of Sciences, 02-109 Warsaw, Poland; bstankiewicz@ibib.waw.pl (B.S.);; 3 Polish Medical Air Rescue, 01-934 Warsaw, Poland

**Keywords:** anaesthetic protocol, multimodal anaesthesia, opioid consumption, opioid harms, opioid-sparing, opioids, paediatric anaesthesia, pain management, strategies reducing opioid requirements

## Abstract

**Background**: Opioids remain the most effective component of systemic analgesia and are considered safe and beneficial when administered at the lowest effective dose. Nevertheless, their potential adverse effects may diminish the quality of the postoperative period or, in some cases, lead to life-threatening complications. This analysis examines whether the mandatory implementation of a standardised protocol offers opioid-sparing potential. **Methods**: In this single-centre retrospective cohort study, intraoperative opioid consumption during laparoscopic appendectomy was compared between patients anaesthetised according to a standardised protocol (*n*_1_ = 132) and those managed at clinicians’ discretion in line with good medical practice (*n*_2_ = 212). Length of hospital stay and use of intraoperative non-opioid analgesics were also assessed. **Results**: The total fentanyl dose administered during anaesthesia was significantly lower in the standardised protocol cohort compared to the cohort without a protocol: 3.13 μg·kg^−1^ (IQR: 2.98–4.08) vs. 5.19 μg·kg^−1^ (IQR: 3.89–6.67), *p* < 0.001. In the protocol cohort, the percentage of patients who received acetaminophen and metamizole was significantly higher—increasing by 57% and 23%, respectively (*p* < 0.001). No significant inter-cohort difference was observed in terms of length of hospital stay. **Conclusions**: The use of a mandatory anaesthetic protocol based on a multimodal approach had an opioid-sparing effect in children undergoing laparoscopic appendectomy. This retrospective analysis was approved by the Ethics Committee of the Medical University of Warsaw (identifier: AKBE/118/2025; date of acceptance: 12 May 2025), and the primary trial was registered in the U.S. National Library of Medicine Clinical Trials Registry (registration number: NCT05238506; date of first registration: 14 February 2022).

## 1. Background

The most versatile and comprehensive form of general anaesthesia consists of a triad: hypnosis, analgesia, and muscle relaxation. The contemporary approach to pain management requires the use of non-opioid analgesics, opioids, adjuvants, and, when feasible, regional anaesthesia [1,2,3]. The multimodal approach has the well-established benefit of providing effective analgesia while minimising the risk of side effects [1,2,3]. Aside from regional anaesthesia techniques—which are unfortunately not applicable to all types of surgery or all patient groups—opioids remain the most effective group of analgesic drugs. This high potency comes at the cost of potential adverse effects, some of which can be distressing or even life-threatening, including respiratory depression, excessive sedation, hallucinations, delirium, nausea and vomiting, constipation, urinary retention, pruritus, and an increased risk of opioid misuse in the long term. Additionally, opioids can cause serotonin syndrome, immunosuppressive effects (such as increased susceptibility to infections and potential cancer progression), and the development of tolerance or opioid-induced hyperalgesia [4]. Despite these risks, opioid-free anaesthesia in adults has been shown to be even more dangerous [5,6]; a study by Beloeil et al. [5] was terminated prematurely after five cases of severe bradycardia occurred in the group who were not receiving opioids, three of which progressed to asystole. Moreover, this group experienced a higher incidence of hypoxaemia within 48 h post-extubation.

However, in the paediatric population, data are less consistent, and there is even some evidence supporting the safe and successful utilisation of opioid-free anaesthesia regimens [7]. Nevertheless, while acknowledging the significant role of opioids, anaesthesiologists should administer them at the minimal necessary doses to reduce the incidence of undesirable sequelae. The most effective strategies for reducing opioid requirements include multimodal analgesia, incorporating regional anaesthesia when feasible, and enhanced recovery pathways [1,2,3,8]. The study purpose was to assess whether exposure to a standardised anaesthetic protocol was associated with lower intraoperative opioid consumption compared with anaesthesia managed at the discretion of individual anaesthetists within accepted clinical practice.

## 2. Methods

### 2.1. Study Design

This study was conducted at a single teaching hospital—the University Clinical Centre of the Medical University of Warsaw, Warsaw, Poland. Patients were enrolled between 12 March 2022 and 8 August 2023.

This study was conducted in accordance with the Declaration of Helsinki, which allows secondary research to be performed using stored data when it is impossible or impracticable to obtain individual consent, provided the project has been reviewed and approved by a research ethics committee. In compliance with current Polish regulations, the Ethics Committee of the Medical University of Warsaw, Warsaw, Poland was notified of the retrospective analysis and granted its approval on 12 May 2025 (identifier: AKBE/118/2025). The Committee determined that the study, which involved a retrospective review of former patients’ medical records, did not constitute a medical experiment and therefore required only formal notification. All data were fully anonymised and are presented as population-level findings, ensuring that no individual could be identified; accordingly, the requirement for patient informed consent was waived. For the prospective trial [9], written informed consent was obtained from all participants aged ≥16 years and from the parents or legal guardians of younger participants.

The primary study [9] was prospective and approved by the Ethics Committee of the Medical University of Warsaw, Warsaw, Poland on 13 December 2021 (KB/204/2021). The trial was registered in the U.S. National Library of Medicine ClinicalTrials.gov database (identifier: NCT05238506), with the date of first registration being 14 February 2022.

In this retrospective cohort study, patients were divided into two exposure groups: the “With Protocol” group, which was exposed to the standardised anaesthesia protocol implemented during the original clinical trial [9], and the “Without Protocol” group, which was exposed to standard anaesthetic care in accordance with good clinical practice, but without adherence to a predefined protocol. The primary objective was to compare intraoperative opioid consumption between the two groups. Secondary outcomes included the use of intraoperative non-opioid analgesics and the length of hospitalisation following the completion of anaesthesia.

All participants underwent the same surgical procedure in the same setting and period of time.

The assignment of patients to each group depended on the anaesthetists’ work schedule: when an anaesthesiologist from the primary study team was on duty, the patient was assessed according to the eligibility criteria and included in the primary study if appropriate, thereby forming the “With Protocol” cohort. When the anaesthesiologist responsible for the case was not a member of the study team, the patient could not be recruited; these patients were later included in the “Without Protocol” cohort.

The primary study team was obligated to enrol every patient who did not meet the trial’s exclusion criteria. Patients who could not be included in the original trial due to objective issues (e.g., language barriers or refusal of consent by a legal guardian) were assigned to the “Without Protocol” cohort and anaesthetised without the standardised protocol. However, this cohort primarily consisted of patients managed by anaesthesiologists who were not part of the study team.

As acute appendicitis is an emergency and the decision to operate is made spontaneously, independent of the anaesthetist’s schedule, group allocation was not controlled by the investigators and appeared unrelated to patients’ medical characteristics, thereby minimising the risk of selection bias.

Anaesthesiologists responsible for patients in the “Without Protocol” group were unaware that their practice would later be analysed and, therefore, retained full autonomy in all clinical decisions.

### 2.2. Participants

To eliminate differences in baseline demographic and clinical characteristics between the compared cohorts, the inclusion criteria matched those of the primary study. Accordingly, only children aged 18 months to 18 years who were admitted for emergency laparoscopic appendectomy and classified as having an American Society of Anesthesiologists (ASA) physical status of 1E, 2E, or 3E were included. Medical archives were screened to identify patients who underwent surgery during the same time period.

### 2.3. Exposure Groups

**Figure 1:**
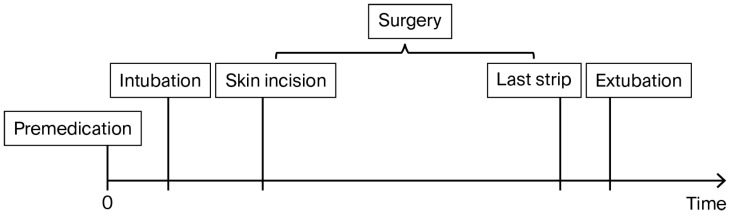
Study timeline diagram.

In this study, two exposure groups were compared: one anaesthetised in accordance with general good clinical practice but without a prespecified mandatory protocol (labelled “Without Protocol”), and the other managed according to an explicit, standardised anaesthesia protocol (labelled “With Protocol”). Both groups of patients were managed using a multimodal approach, in accordance with American and Polish pain management guidelines [1,2,3]. The study timeline is presented in Figure 1.

The “Without Protocol” cohort consisted of patients anaesthetised by paediatric anaesthesiologists working in the Department of Paediatric Anaesthesiology and Intensive Care at the University Clinical Centre of the Medical University of Warsaw. This is a teaching hospital dedicated exclusively to caring for children. The department comprises 28 physicians who provide anaesthesia for a wide range of procedures, including cardiac surgery, neurosurgery, general paediatric surgery, orthopaedic surgery, laryngology, catheterisation laboratory procedures, and endoscopic interventions. The team is also responsible for managing the intensive care unit, cardiothoracic intensive care unit, and post-anaesthesia care unit (PACU). Patients in the “Without Protocol” group were anaesthetised by physicians who were not involved in the original trial [9], and whose clinical decisions were based on individual professional experience yet remained within the framework of accepted medical standards. Specifically, these practices adhered to both national and international guidelines on pain management and anaesthesiology [1,2,3], as well as the national legal regulations outlined in the Notice of the Minister of Health on the Organisational Standards of Healthcare in the Field of Anaesthesiology and Intensive Care (Journal of Laws of the Republic of Poland). Accordingly, anaesthesiologists retained the discretion to apply their clinical judgement—for example, by selecting any dose within a recommended range or omitting interventions supported by weak evidence—while remaining compliant with procedural and legal standards.

Decisions such as whether to administer premedication with midazolam, the type and dose of muscle relaxant to use (see below), the choice of volatile anaesthetic (sevoflurane vs. desflurane), and the timing and selection of simple analgesics were all left to the discretion of the individual anaesthesiologist. Furthermore, in contrast to standard practice for adults, not all cases of acute appendicitis in this cohort were classified as emergencies (E), and following preanaesthetic evaluation, not all patients were managed with full-stomach precautions.

In children, the recommended induction dose of fentanyl ranges from 2 to 5 μg·kg^−1^, providing physicians with considerable flexibility while remaining within the bounds of good medical practice. In this cohort, additional doses were administered based on haemodynamic changes, pupil reactivity, and signs such as sweating; however, no strict thresholds were defined, and the dose decision was once again left to individual clinical judgement.

The “With Protocol” cohort comprised all participants from both original arms of the trial conducted by Kaszyński et al. [9], who were combined into a single group for the purposes of this analysis. In the primary study, no opioid-sparing effect was demonstrated following intravenous lidocaine infusion in children undergoing laparoscopic appendectomy—no difference was found between lidocaine and placebo (normal saline) for this indication. Therefore, in the present retrospective cohort analysis, lidocaine played no role in the study methodology.

Patients in this cohort were monitored and anaesthetised in accordance with a mandatory anaesthesia protocol, as described below.

Anaesthesia Protocol for the “With Protocol”. (The following description of the anaesthesia protocol is identical to that published in the original trial by Kaszyński et al. [9] and is reproduced here for clarity and consistency. This repetition is intentional and necessary to ensure accurate representation of the methods used, and to avoid any misinterpretation. Appropriate citation has been provided to acknowledge the original source.)

The peripheral intravenous catheter was inserted in the Emergency Department or in the Surgery Ward when obtaining blood samples. No local anaesthetics were used.

Due to the primary diagnosis of acute appendicitis, all cases were classified as emergencies (E) and all patients were treated as if they had a “full stomach” and were therefore at risk of pulmonary aspiration. Rapid sequence intubation was performed with a high dose of rocuronium without applying cricoid pressure (the Sellick manoeuvre).

Upon admission to the operating wing, intravenous midazolam at 0.05 mg kg^−1^ was administered for anxiolysis. The patient was then transferred to the operating theatre, where their vital signs were captured. In both groups, identical syringes containing an unidentifiable substance (1% lidocaine or normal saline) were connected, and a bolus of 0.15 mL kg^−1^ was administered over five minutes. Induction of anaesthesia was achieved with IV propofol 4 mg kg^−1^, fentanyl 3 µg kg^−1^ and rocuronium 1.0–1.2 mg kg^−1^. Five minutes after tracheal intubation, the first blood sample was collected into a blood collection tube, and the infusion of the masked solution was initiated.

This was followed by intravenous acetaminophen 15 mg kg^−1^ and metamizole 15 mg kg^−1^. Anaesthesia was maintained with sevoflurane. The minimum alveolar concentration of the volatile agent was adjusted to maintain the bispectral index (BIS) near the target of 45.

Additional fentanyl doses of 1 µg kg^−1^ were given when the increase in heart rate (HR) or blood pressure (BP) exceeded 20% of baseline readings.

The second blood sample was collected into a blood collection tube immediately after the end of surgery (when the last dressing was applied) and before the endotracheal tube was removed. After extubation, the children were transferred to the postanaesthesia care unit (PACU).

Haemodynamic and respiratory parameters were continuously monitored, recorded, and automatically stored in the study database for future analysis.

## 3. Study Outcomes

Data for both cohorts were obtained in the operating wing. In the “With Protocol” group, information was gathered prospectively as part of the original study, while for the “Without Protocol” group, it was retrieved retrospectively from individual medical records, operating wing documentation, and the hospital server, which stores outputs from cardiomonitors and anaesthesia machines.

### 3.1. Primary Outcome

Intraoperative opioid consumption is assessed by comparing the total amount of fentanyl administered—from the induction of anaesthesia to admission to the PACU—measured in micrograms per kilogram of body weight.

### 3.2. Secondary Outcomes

Length of stay (LOS): Defined as the duration of hospitalisation following the completion of anaesthesia, measured from the time of extubation to discharge from the hospital.

Use of intraoperative non-opioid analgesics: Defined as the percentage of patients in each group who received acetaminophen and metamizole, reported separately for each substance.

### 3.3. Statistical Analysis

Quantitative data were assessed for normal distribution using the Shapiro–Wilk test. Due to non-normal data distribution, they are reported as median with interquartile range (IQR) (Table 1 and Table 2). The Mann–Whitney U test was utilised to compare the “With Protocol” and “Without Protocol” group data (on age, weight, analgesic doses, and time periods) for the same reason.

Categorical data—the sets of counts and proportions—are expressed as the number of participants (e.g., those who received a specific analgesic) and the corresponding percentage of the group (Table 1 and Table 2). Inter-group differences in the proportions received for a given category were assessed with the χ^2^ test.

A *p*-value < 0.05 was considered to be statistically significant, and all analyses were conducted using STATISTICA (StatSoft, Inc. (Tulsa, OK, USA) (2011). STATISTICA (data analysis software system), version 10. www.statsoft.com).

## 4. Results

### 4.1. Study Population

Between 12 March 2022 and 8 August 2023, a total of 362 patients underwent an eligibility assessment for the primary study [9]. Of these, 132 were anaesthetised using the standard trial protocol, 212 were anaesthetised concurrently outside the study, and 18 patients either did not meet the inclusion criteria or had severely incomplete data.

Baseline demographic and clinical characteristics were comparable between cohorts and are summarised in Table 1.

### 4.2. Perioperative Clinical Data

Perioperative clinical data comparing the “With Protocol” and “Without Protocol” groups are presented in Table 2.

### 4.3. Primary Outcome Results (Figure 2)

The total dose of fentanyl administered during anaesthesia was significantly lower in the “With Protocol” group—3.13 μg·kg^−1^ (IQR: 2.98–4.08)—compared to the “Without Protocol” group—5.19 μg·kg^−1^ (IQR: 3.89–6.67), *p* < 0.001.

**Figure 2:**
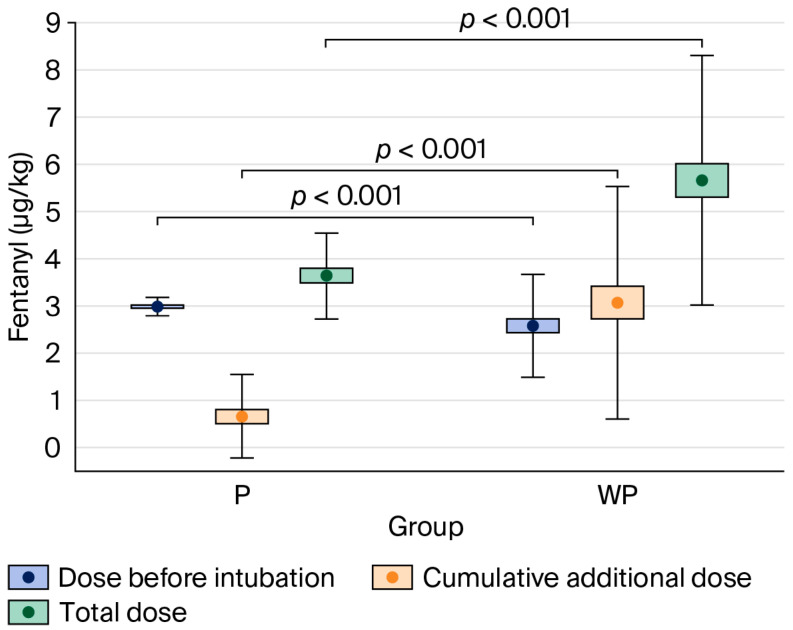
Comparison of fentanyl dose per body mass administered in the “With Protocol” (P) and “Without Protocol” (WP) groups before intubation and intraoperatively (cumulative additional dose), and the total dose. The data are mean ± 95% CI (box) and mean ± SD (whiskers). CI—confidence interval; SD—standard deviation.

The percentage of patients who received an additional dose of fentanyl after the start of surgery was significantly lower in the “With Protocol” group (45%) compared to the “Without Protocol” group (90%), *p* < 0.001.

### 4.4. Secondary Outcomes Results

#### 4.4.1. Length of Stay (LOS)

There was no significant difference between the “With Protocol” and “Without Protocol” groups in the time from extubation to discharge from the hospital: 4.38 days (IQR: 3.67–6.62) and 4.59 days (IQR: 3.58–6.82), respectively; *p* = 0.295.

#### 4.4.2. Use of Intraoperative Non-Opioid Analgesics (Figure 3)

The percentage of patients who received acetaminophen and metamizole was significantly higher in the “With Protocol” than the “Without Protocol” group (acetaminophen: 100% vs. 43%, *p* < 0.001; metamizole: 100% vs. 77%, *p* < 0.001) (see Table 2).

**Figure 3:**
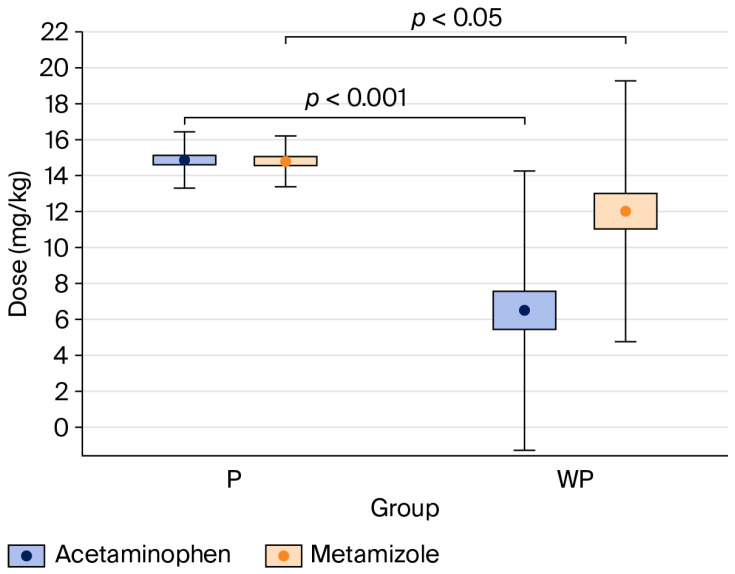
Comparison of acetaminophen and metamizole doses per body weight between the “With Protocol” (P) and “Without Protocol” (WP) groups. The data are mean ± 95% CI (box) and mean ± SD (whiskers). CI—confidence interval; SD—standard deviation.

Compared to the “Without Protocol” group, patients in the “With Protocol” group received significantly higher doses of acetaminophen (*p* < 0.001) and metamizole (*p* < 0.05), expressed in mg·kg^−1^ (see Table 2).

## 5. Discussion

According to the Declaration of Montreal, access to pain management is a fundamental human right. The first article of the declaration emphasizes “the right of all people to have access to pain management without discrimination, in particular, on the basis of age, sex, gender, medical diagnosis, race, religion, culture, marital status, or political or other opinion” [10]. However, children represent a particularly vulnerable patient population and are especially at risk of receiving inadequate analgesia [11]. Undertreated pain in this group may lead to long-term consequences, including chronic pain, anxiety, and depressive disorders [11]; therefore, special attention must be given to this issue. In our study, we focused on laparoscopic appendectomy—a procedure associated with moderate to severe acute pain—which warrants the use of opioids according to the World Health Organization and contemporary pain management guidelines [1,2,3].

Although opioids have been used in perioperative and periprocedural medicine for thousands of years [12], their role continues to be critically evaluated, with efforts ongoing to identify safe and effective methods of administration. It is now well established that both opioid-free and high-dose-opioid anaesthesia can expose patients to significant risks [5,6,12], and in some cases, the complete avoidance of opioids may critically compromise patient safety [5]. However, with future advancements—such as the identification of genetic markers associated with addiction risk—it may become possible to safely tailor opioid-free regimens for selected high-risk individuals [12]. With today’s high standards of patient monitoring and the implementation of opioid-sparing strategies in both analgesia and anaesthesia, it is widely accepted that opioids should be administered at the lowest effective doses and for the shortest possible duration.

To achieve this goal, the concept of multimodal analgesia was introduced, evaluated, and continues to evolve. All contemporary pain management guidelines [1,2,3,11] support this approach, as it ensures adequate anaesthesia while reducing the required dose of each component in the therapeutic regimen to minimise the risk of drug-specific side effects.

In the present study, the implementation of a strict anaesthesia protocol proved superior in reducing opioid doses compared to the absence of any standardised approach, in which decisions were made by individual physicians guided by their own knowledge, experience, and clinical judgement—within the bounds of accepted medical practice. The standardised protocol was based on a multimodal approach, which included the administration of a relatively high initial dose of fentanyl, along with additional rescue doses if predefined thresholds for HR or BP were exceeded. Patients in the “With Protocol” group received a significantly higher dose of fentanyl during the induction of anaesthesia—3.00 μg·kg^−1^ (IQR: 2.90–3.12)—compared to the “Without Protocol” group—2.35 μg·kg^−1^ (IQR: 1.89–2.94), *p* < 0.001.

In contrast, the percentage of patients who required an additional dose of fentanyl after the start of surgery was significantly lower in the “With Protocol” (45%) compared to the “Without Protocol” group (90%), *p* < 0.001.

Ultimately, likely due to the strict criteria governing rescue fentanyl dosing and the potential pre-emptive analgesic effect resulting from the higher fentanyl dose administered during induction, the total amount of fentanyl administered intraoperatively was significantly lower in the “With Protocol” group compared to the “Without Protocol” group: 3.13 μg·kg^−1^ (IQR: 2.98–4.08) vs. 5.19 μg·kg^−1^ (IQR: 3.89–6.67); *p* < 0.001. This difference may also be attributed to the consistent use of non-opioid analgesics in the “With Protocol” group, compared to significantly fewer patients receiving them in the “Without Protocol” group (see Table 2). Regardless of the underlying cause, the marked difference in overall opioid exposure between the two groups warrants future clinical trials to determine the relative contribution of each factor.

Although the present analysis supports the introduction of an anaesthesia protocol to reduce intraoperative opioid use, it demonstrated no effect on the length of hospital stay following anaesthesia. Therefore, it cannot be concluded that a lower intraoperative opioid dose shortens the time required for discharge readiness, as suggested by some studies [8]. Nevertheless, it is plausible that reduced opioid use may improve the quality of the early postoperative period by decreasing the incidence of opioid-specific side effects, such as respiratory depression, excessive sedation, hallucinations, delirium, nausea and vomiting, constipation, urinary retention, and pruritus. However, this aspect was beyond the remit of our analysis, which focused predominantly on the intraoperative period.

Finally, while it is well established that both the multimodal approach and ERAS (Enhanced Recovery After Surgery) protocols reduce opioid utilisation—and their effectiveness has been demonstrated in numerous studies [1,2,3,8,13,14,15]—our analysis addresses a different aspect. Specifically, it assesses whether, in a setting where multimodal strategies are already applied according to internal guidelines and where all medical staff are trained, have attended dedicated lectures, passed an exam, and are certified—there is still additional benefit in mandating explicit anaesthesia protocols.

In conclusion, this possibility is at least worth serious consideration. It also raises a broader question: Can an individual physician consistently make better decisions than a structured algorithm, and if so, should we begin to reconsider and redefine the role of the human element in the therapeutic process? However, this discussion lies beyond the scope of the present paper.

### Study Limitations

The main limitation of this study is that the observations are obtained from a single centre; however, the Department of Paediatric Anaesthesiology and Intensive Care at the Medical University of Warsaw University Clinical Centre is responsible for training anaesthesiologists during their specialist education in the Masovian Province and is therefore likely to be representative of the region. A similar multi-centre study would provide more robust evidence.

A second limitation of the trial is its retrospective nature. On the other hand, the anaesthesiologists responsible for patients in the “Without Protocol” group were unaware that their practice would later be analysed, and so their clinical decisions were not influenced by the awareness of being observed, allowing for the collection of data that reflect genuine, unaffected clinical habits.

The third limitation of this study is that it focuses exclusively on the period of general anaesthesia. Although the quality of postoperative recovery is an important consideration, it was beyond the scope of this analysis. In the SOFA trial [16], compared with standard anaesthesia, opioid-free anaesthesia led to a statistically significant but not clinically meaningful improvement in recovery quality following major elective surgery. Based on these findings, it is reasonable to assume that the two similar anaesthetic protocols analysed in this study would likely have an even smaller impact on the postoperative period. Nevertheless, this hypothesis warrants evaluation in a dedicated prospective trial.

The fourth limitation is that this study focuses solely on patients undergoing laparoscopic appendectomy. Further trials are needed to assess whether the opioid-sparing effects of a mandatory anaesthesia protocol can be replicated in other patient cohorts.

## 6. Conclusions

The use of a mandatory anaesthetic protocol, based on a multimodal approach that included a substantial opioid dose administered during induction, significantly reduced the total intraoperative fentanyl dose in children undergoing laparoscopic appendectomy.

These findings support the consideration of implementing standardised anaesthetic protocols.

## Figures and Tables

**Table 1:** Patient characteristics in each cohort.

Variable	Group (*n* = 132) With Protocol	Group (*n* = 212) Without Protocol
**Age**		
(years) Median (IQR)	11.79 (8.58–14.38)	11.45 (8.18–14.06)
**Sex**		
**Male**		
*n* (%)	74 (56)	136 (64)
**Female**		
*n* (%)	58 (44)	76 (36)
**Weight**		
(kg) Median (IQR)	43 (29.5–56.5)	41 (28–58)
**ASA**		
(1E/2E/3E)	84/47/1	107/100/5

Quantitative non-normally distributed data were expressed as median (IQR, interquartile range) and compared using the Mann–Whitney U test. Categorical data, including sets of counts (*n*) and proportions (in %), were compared by the χ^2^ test.

**Table 2:** Perioperative clinical data.

Variable	With Protocol	Without Protocol	*p*-Value
	*n* = 132	*n* = 212	
**Duration of surgery**			
(min) Median (IQR)	54.96 (39.96–69.96)	60 (45–80)	<0.05
**Duration of anaesthesia**			
(min) Median (IQR)	87 (69.96–103.47)	83 (70–109)	0.912
**Time from the end of surgery (“last strip”) to extubation**			
(min) Median (IQR)	12 (9.96–15.48)	11 (5–16)	<0.005
**Time from extubation to discharge**			
(days) Median (IQR)	4.38 (3.67–6.62)	4.59 (3.58–6.82)	0.295
**Fentanyl before intubation**			
(µg) Median (IQR)	130 (90–165)	100 (80–100)	<0.001
(µg kg^−1^) Median (IQR)	3 (2.9–3.12)	2.35 (1.89–2.94)	<0.001
**Cumulative dose of additional fentanyl**			
(µg) Median (IQR)	0 (0–42.5)	100 (50–195)	<0.001
(µg kg^−1^) Median (IQR)	0 (0–1.05)	2.5 (1.61–4)	<0.001
**Total fentanyl dose**			
(µg) Median (IQR)	150 (105–200)	200 (150–300)	<0.001
(µg kg^−1^) Median (IQR)	3.13 (2.98–4.08)	5.19 (3.89–6.67)	<0.001
**Number of patients who received additional fentanyl dose**			
*n* (%)	59 (45)	191 (90)	<0.001
**Total use of intraoperative non-opioid analgesics**			
**Acetaminophen (paracetamol)**			
(mg) Median (IQR)	640 (450–900)	0 (0–500)	<0.001
(mg kg^−1^) Median (IQR)	15 (9.09–20)	0 (0–14.89)	<0.001
**Number of patients who received acetaminophen (paracetamol)**			
*n* (%)	132 (100)	91 (43)	<0.001
**Metamizole**			
(mg) Median (IQR)	625 (450–850)	500 (200–1000)	<0.05
(mg kg^−1^) Median (IQR)	15 (9.09–20)	14.71 (10–15.91)	<0.05
**Number of patients who received metamizole**			
*n* (%)	132 (100)	163 (77)	<0.001

Quantitative non-normally distributed data were expressed as the median (IQR, interquartile range) and compared using the Mann–Whitney U test. Categorical data, including sets of counts (*n*) and proportions (in %), were compared by the χ^2^ test.

## Data Availability

The datasets used and/or analysed during the current study are available from the corresponding author upon reasonable request.

## Abbreviations

ASA	American Society of Anesthesiologists
BIS	Bispectral Index
BP	Blood Pressure
CI	Confidence Interval
ERAS	Enhanced Recovery After Surgery
HR	Heart Rate
IQR	Interquartile Range
PACU	Post-anaesthesia Care Unit
SD	Standard Deviation
